# Comparison of the Mandibular Bone Densitometry Measurement Between Normal, Osteopenic and Osteoporotic Postmenopausal Women

**Published:** 2013-05

**Authors:** Leila Khojastehpour, Sara Mogharrabi, Mohammad Hossein Dabbaghmanesh, Nariman Iraji Nasrabadi

**Affiliations:** 1 Associate Professor, Department of Oral and Maxillofacial Radiology, School of Dentistry, Shiraz University of Medical Sciences, Shiraz, Iran; 2 Postgraduate Student of Prosthodontics, Department of Prosthodontics, School of Dentistry, Shiraz University of Medical Sciences, Shiraz, Iran; 3 Professor, Endocrine and metabolism Research Center, Shiraz University of Medical Sciences, Shiraz, Iran; 4 Dentist, Member of Student Research Committee, School of Dentistry, Shiraz University of Medical Sciences, Shiraz, Iran

**Keywords:** Postmenopause women; Osteopenia; Osteoporosis; Panoramic Radiograph; Digital; Mandible; Density

## Abstract

**Objective:** This study was conducted to compare the mandibular bone density between postmenopausal women with normal skeletal bone mass density (BMD) and those with low skeletal BMD using digital panoramic radiographs.

**Materials and Methods:** One hundred fifteen postmenopausal women were divided into normal and osteoporotic/osteopenic groups. Digital panoramic radiographs were prepared using Digora PCT Sorodex equipment and Promax panoramic X-ray unit (Planmeca, Helsinki, Finland, Kvp=68 and mA=9). The mandibular bone density of an area (approximately 4×4 mm), exactly near the distal edge of the right mental foramen was determined in digital panoramic radiographs using Digora for Windows (DfW) Software.

**Results:** There was no statistically significant difference in mandibular bone density between the normal and osteoporotic/osteopenic participants (P >0.05). Mandibular bone density was not statistically different in normal and osteoporotic individuals with SBMD or FBMD T-score -2.5 (P >0.05). Density of the region of interest differed significantly between the normal and the osteoporotic group with SBMD and FBMD T-score -2.5 (P <0.05). The same results also gained in women who were osteoporotic only in the femoral region (P <0.05).

**Conclusion: **Mandibular bone density in subjects with low BMD was related to FBMD. So, digital panoramic radiographs could be beneficial in the diagnosis of postmenopausal women who are at risk of osteoporosis.

## Introduction

Osteoporosis is a systemic skeletal disease characterized by decreased bone density and microstructural deterioration of bone tissue [1]. Patients are disabled by fractures leading to substantial morbidity, increased medical costs and a remarkably poor quality of life in the elderly [2]. 

Osteoporosis risk factors include smoking, alcohol consumption, physical inactivity, genetics, low calcium intake, drug therapy with glucocorticoids, antiepileptic and anticoagulant drugs and diseases which affect bone metabolism [3]. The hormonal changes that accompany menopause are the most important cause of decreased bone mass in women [4]. Identifying asymptomatic individuals at risk of fractures is important to control the increase in morbidity, mortality and medical costs worldwide [5].The imaging techniques used for diagnosing osteoporosis are based on the measurement of bone mineral density and bone mineral content. Dual-energy X-ray absorptiometry (DXA) is currently the most widely used technique and it is considered as the ‘gold standard’ in diagnostic techniques, enabling the diagnosis and monitoring of the bone mass loss [6]. 

DXA measures bone density as “area density” in units of gram/cm^2^. This technology is used to measure BMD in some central (hip and spine) and peripheral sites (radius). 

In order to facilitate the interpretation of the examination, results include the normal range of BMD for women aged 20 to 80 years and the patient’s actual BMD. 

The T-score represents the difference (standard deviation) from the peak bone mass for the population. According to WHO classification, a BMD of more than 1 and 2.5 standard deviation below this peak represent osteopenia and osteoporosis, respectively [7]. 

Since panoramic radiographs are among the most frequent X-ray images available in the population, several studies have been done on possible usefulness of it as a screening tool for detection of various health problems that affect alveolar bones including osteoporosis. Most of these studies have focused on the thickness and integrity of the inferior border of the mandible. 

The effect of osteoporosis on alveolar bone loss and tubercular bone pattern has also been evaluated [8]. 

Rapid development of digital imaging has affected dentistry as well, and various digital panoramic equipments and their software provide more facilities and information that were not available directly in film based radiography [9]. 

The aim of the present study was to compare mandibular density measurements of normal, osteopenic and osteoporotic postmenopausal women in digital panoramic radiographs.

## MATHERIALS AND METHODS

One hundred fifteen healthy non-smoker postmenopausal Iranian women at the age of 40-70 years were recruited for this cross-sectional study. 

They were selected among those who were referred to Namazi Hospital of Shiraz University of Medical Sciences for evaluation of osteoporosis from May 2008 to June 2009. They were all in a natural menopause phase. Corticosteroid therapy, alcoholism and systemic diseases that would affect bone metabolism such as hyperpara-thyroidism, hypoparathyroidism, Paget‟s disease, thyrotoxicosis, malabsorption, liver diseases and cancers with bone metastasis were considered as exclusion criteria. All subjects participated voluntarily and informed consent was obtained from all participants. 

This study was approved by the “Medical Research Ethics Committee” of Shiraz University of Medical Sciences. DXA scans were performed (DXA, LUNAR DPX IQ) in the neck of the femur and the spine (L2-L4). 

Based on their BMD results and according to the World Health Organization (WHO) criteria, subjects were classified as normal (T-score of >-1.0), osteopenic (T-score of -1 to -2.5) or osteoporotic (T-score of <-2.5). An oral digital panoramic radiograph was prepared for each participant using Digora PCT Sorodex equipments and Promax panoramic X-ray unit (Planmeca, Helsinki, Finland, Kvp=68 and mA=9). The position of the head was standardized as much as possible. 

**Fig1:**
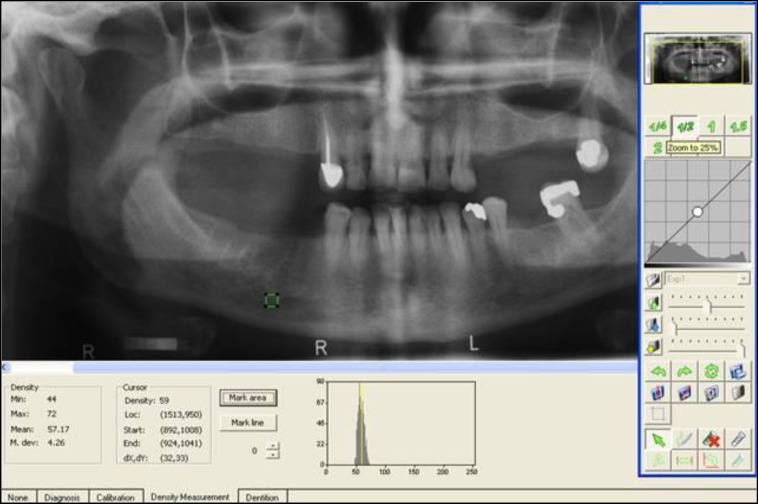
Analyzing a digital panoramic radiograph using Digora software

An 8 step aluminum step wedge was used for density calibration. The interval between DXA examination and taking radiographs was not longer than 2 weeks. An area with approximately 4×4 mm dimension just near the distal edge of the right mental foramen in the digital panoramic radiograph was selected and DfW was calculated and reported maximum, minimum and mean density (Fig1). Whenever impossible to find the right foramen, the left foramen was used. A radiologist who was expert in using DfW software did all measurements twice with a one week interval. For evaluation of obtained data of mandibular bone density between normal and osteoporotic/osteopenic groups, femoral bone mass density (FBMD) and spinal bone mass density (SBMD) values were considered both separately and together. SPSS 17 for Windows (SPSS Inc., Chicago, Ill, USA) was used for statistical analysis. 

Intraclass correlation coefficient (ICC) was used to assess the reliability of the observer measurements. Independent t-test was used to compare mandibular bone density in normal and osteoporotic/osteopenic groups.

## Results

 Out of 115 recruited postmenopausal women, 11 women who had incomplete records were excluded. So, 104 women were considered for analysis. Their mean age was 54.88 years (SD=5.89). Forty five subjects (43.26%) who had normal BMD records in both sites were classified as the control group. 

A total of 59 (56.73%) subjects were included in the osteopenic/osteoporotic group as they had a FBMD or SBMD T-score less than –l.

Among the osteoporotic/osteopenic group, 14 women were osteoporotic in both lumbar spine and femoral neck regions.

**Table 1:** Comparison of mandibular bone density in normal and osteoporotic/osteopenic

**Alveolar Bone Density**	**FBMD or SBMD T-score ≤-1** **(Low Skeletal BMD) N=56**		**Normal** **N=41**		**P value**
	**Mean **	**SE**	**Mean **	**SE**	
**Minimum**	41.75	2.57	42.63	3.22	0.829
**Maximum**	65.88	3.22	73.71	4.24	0.139
**Mean**	52.01	2.43	52.01	2.94	0.393

**Table 2:** Comparison of mandibular bone density in normal and osteoporotic

**Alveolar Bone Density**	**FBMD or SBMD T-score** **≤-2.5 (Osteoporosis) N=45**		**Normal** **N=41**		**P value**
	**Mean**	**SE**	**Mean**	**SE**	
**Minimum**	42.24	3.08	42.63	3.22	0.956
**Maximum**	65.67	3.87	73.71	4.24	0.211
**Mean**	52.23	3.59	52.01	2.94	0.554

* Significant P value <0.05

**Table 3:** Comparison of mandibular bone density in normal and osteoporotic (SBMD and FBMD T-score ≤ -2.5) groups

**Alveolar Bone Density**	**FBMD and SBMD T-score** **≤-2.5 (Osteoporosis)** **N=14**		**Normal** **N=41**		**P value**
	**Mean**	**SE**	**Mean**	**SE**	
**Minimum**	34.29	3.23	42.63	3.22	0.287
**Maximum**	55.86	4.52	73.71	4.24	0.033*
**Mean**	42.86	3.79	52.01	2.94	0.074

* Significant P value <0.05

**Table 4:** Comparison of mandibular bone density in normal and osteoporotic (FBMD T-score ≤ -2.5) groups

**Alveolar Bone Density**	**FBMD T-score ≤-2.5** **N=19**		**Normal** **N=41**		**P value**
	**Mean **	**SE**	**Mean **	**SE**	
**Minimum**	35.84	4.76	42.63	3.22	0.242
**Maximum**	57.21	5.67	73.71	4.24	0.028*
**Mean**	44.66	5.37	52.01	2.94	0.086

* Significant P value <0.05

Records of four normal participants were omitted due to difficulties in recognition of the mental foramen. As demonstrated in Table 1, there is no statistically significant difference in mandibular bone density between the normal and osteoporotic/osteopenic participants with SBMD or FBMD T-score ≤ -1 (*P* >0.05). 

Table 2 indicates that mandibular bone density is not statistically different in normal and osteoporotic individuals with SBMD or FBMD T-score ≤ -2.5 (*P* >0.05). Density of the region of interest differs significantly between the normal and the osteoporotic group with SBMD and FBMD T-Score ≤ -2.5 (*P* <0.05) (Table 3). 

The same results also gained in women who were osteoporotic only in the femoral region (FBMD T-Score ≤ -2.5) (Table 4).

## Discussion

Panoramic radiography as a screening tool for low bone density has been discussed for decades, but we did not use special software to calculate gray scale and bone density in our study. We used Digora for Windows (DfW) software that is available in every radiology clinic and provides digital panoramic radiographs by Panoramic Sorodex devices. BMD testing of all postmenopausal women at risk requires extensive facilities, time and high costs. Therefore, we tried to find a less expensive method for prediction of osteoporosis at large scale that does not require a special clinic and is also beneficial in developing countries. This study was carried out to see if there is any relation between mandibular density measurement in panoramic radiograph and BMD. The difference however, was found only in maximum mandibular bone density value between the control and osteoporotic group. In addition, this difference was limited to the subjects who were osteoporotic in the femoral region alone or in combination with the spinal region (FBMD T-Score ≤ -2.5 or both FBMD and SBMD T-Score ≤ -2.5). 

We had hypothesized that FBMD is more related to mandibular bone density than SBMD. It could be supported by this fact that there are some studies in which the investigators only assessed the association of femoral osteoporosis with various panoramic radiographic findings in postmenopausal women [10,11]. The results of a study conducted by Taguchi et al. showed that postmenopausal women with femoral osteoporosis may be identified by measuring the mandibular cortical width (MCW) on panoramic radiographs with sufficient diagnostic efficacy [11]. 

In addition, our results were somewhat similar to that of Amorim and coworkers who found an association between the low femoral neck BMD and poor mandibular bone quality as assessed by panoramic radiography in patients receiving dental implants [10]. The ability of oral radiographs in identifying osteoporosis in patients has been assessed in several studies. However, using intraoral radiographs is less popular than panoramic radiographs. Considering bone density measurement, it could be related to necessities of using aluminum step wedge for calibration of density. 

According to the previous studies this procedure is very difficult and time consuming, furthermore step wedge shadow may superimpose on all or some parts of the radiographs. Nackearts et al. reported 9 to 13% data loss for the maxillary and mandibular periapical radiographs respectively [1], they had reported that including an aluminum step wedge on the intraoral radiograph was a challenge and many radiographs could not be measured because the teeth overlapped the step wedge, or the wedge was partially or fully out of the projection filed. 

For avoiding such difficulties in this study we used digital panoramic radiographs. Another point was selecting the appropriate region of interest. Based on previous studies it seems that there is no preferred jaw and dental radiographs of both maxillary and mandibular bone can be used for predicting osteoporosis [12-15]; however, most of the researchers only involve mandibular measurements in their analysis and consider the area of the mandible posterior to the mental foramen as the standard measurement site for jaw bone analysis [1] because it has the lowest inter- and intra-individual variations in anatomical size, shape, bone structure and function [16]. 

This fact that both case and control groups in this study were among those women who visited the BMD assessment ward of Namazi hospital could be considered as a possible limitation of this study as our subjects were volunteers from the community and were not representative of normal postmenopausal women. A Second limitation may be related to deficiency of Digora software tools to create the exact same sized region of interest. However, it is comparable with other studies that reported an average size of the region of interest rather than its exact size [1,11]. 

Nackarta et al. used custom made software and used the region of interest with on average 30×30 pixels for determining the diagnostic accuracy of mandibular and maxillary bone density in detecting osteoporosis. They suggested that although jaw bone density measurements are not an excellent characteristic for osteoporosis, the density of the premolar region reaches a fair diagnostic accuracy [1,17]. 

## CONCLUSION

The results of this study showed that reduced skeletal bone mass is not totally related with mandibular density measurement with DfW software in panoramic radiographs. This could be related to the age of the study group. 

Thus, additional investigation of the relationship between mandibular bone densitometry and osteoporosis in all age groups of women using Digora software is necessary to understand the reliability of digital panoramic radiographs in the prediction or early diagnosis of osteoporosis.
